# Accuracy Analysis of a New Data Processing Method for Landslide Monitoring Based on Unmanned Aerial System Photogrammetry

**DOI:** 10.3390/s23063097

**Published:** 2023-03-14

**Authors:** Ivan Jakopec, Ante Marendić, Igor Grgac

**Affiliations:** 1 Department of Applied Geodesy, Faculty of Geodesy, University of Zagreb, Kačićeva 26, 10000 Zagreb, Croatia; ivan.jakopec@geof.unizg.hr; 2 PNT Tech d.o.o., Bukovački Obronak 26, 10000 Zagreb, Croatia; igrgac.pnttech@gmail.com

**Keywords:** landslide, monitoring, unmanned aerial systems, structure from motion, photogrammetry

## Abstract

One of the most commonly used surveying techniques for landslide monitoring is a photogrammetric survey using an Unmanned Aerial System (UAS), where landslide displacements can be determined by comparing dense point clouds, digital terrain models, and digital orthomosaic maps resulting from different measurement epochs. A new data processing method for calculating landslide displacements based on UAS photogrammetric survey data is presented in this paper, whose main advantage is the fact that it does not require the production of the above-mentioned products, enabling faster and simpler displacement determination. The proposed method is based on matching features between the images from two different UAS photogrammetric surveys and calculating the displacements based only on the comparison of two reconstructed sparse point clouds. The accuracy of the method was analyzed on a test field with simulated displacements and on an active landslide in Croatia. Moreover, the results were compared with the results obtained with a commonly used method based on comparing manually tracked features on orthomosaics from different epochs. Analysis of the test field results using the presented method show the ability to determine displacements with a centimeter level accuracy in ideal conditions even with a flight height of 120 m, and on the Kostanjek landslide with a sub-decimeter level accuracy.

## 1. Introduction

Landslides are one of the world’s major geohazards that can cause great material, economic, social, and human losses [1]. Due to climate changes, natural extremes such as rapid temperature changes, flooding, and high amounts of precipitation in a short period can affect the slope stability in general [2,3,4]. Many landslides were activated in Croatia caused by those processes and resulted in significant material damage [5]. Landslide monitoring can provide vital information for preventing or reducing potential great dangers that landslides cause [6]. As a good example of a landslide monitoring system, a continuous real-time monitoring system installed on landslide Kostanjek, located in the city of Zagreb in Croatia, can be highlighted. The monitoring system acquires information of landslide kinematics to react in time and prevent a potential catastrophe [7,8]. The information collected by the monitoring system is necessary for understanding the landslides behavior and for identifying possible triggering effects [9].

The most common techniques used in landslide monitoring projects are ground-based techniques, including geotechnical, geophysical, and geodetic instruments, and remote sensing techniques [10]. Classical geodetic techniques include measurements using the level, total station, and GNSS instruments [11,12,13]. The main advantage of these techniques is the millimeter level accuracy, and in many studies, those measurements were considered as reference values used to access the validation of other monitoring techniques [11,14,15]. However, monitoring with these techniques does not provide complete information on landslide displacements due to the sparse spatial distribution of surveyed points [16]. Furthermore, remote sensing techniques for landslide monitoring utilize terrestrial, airborne, and spaceborne optical and microwave images, as well as airborne (ALS) and TLS datasets [10]. Remote sensing techniques are practical tools to rapidly obtain spatially distributed information on landslide kinematics [17]. The main advantage of remote sensing techniques is the capability to acquire spatially continuous data with centimeter precision, which can complement conventional ground-based techniques [18]. Those techniques allow the measurements of ground displacements, variations of geotechnical or geophysical parameters, and water level measurements [19], which means that they can improve the knowledge about the development of landslides and their triggering factors [20].

Due to technological developments in the last few decades, the use of Unmanned Aerial Systems (UAS) in the field of Photogrammetry and Remote sensing is constantly growing [21]. The monitoring technique based on UAS photogrammetric surveying has become a valuable technique in the landslide monitoring projects [22,23,24,25]. Using this technique, the monitoring is based on quantifying the topographic changes between products such as dense point cloud, digital elevation model (DEM), digital surface model (DSM), digital terrain model (DTM), and digital orthomosaic [22,23] obtained from different UAS survey epochs and delivered from the Structure from Motion (SfM) and Multi-View Stereo (MVS) image processing algorithm. Among the listed techniques based on UAS photogrammetric surveying, one of the most used techniques is based on image correlation, which detects corresponding features in two images by correlating their intensity values to detect topographic changes. Using this technique on georeferenced orthomosaic images produced from different UAS survey epochs, the horizontal displacement of the landslide can be determined [26,27,28]. Moreover, the widely used technique is DEM of difference (DoD), by which a comparison of digital elevation models obtained from two different UAS survey epochs is performed to obtain landslide displacements in periods between survey epochs [29,30,31]. Many authors use Multiscale Model-to-Model Cloud Comparison (M3C2) for directly comparing two point clouds and conducting change detection with minimal manual processing [31,32,33].

All the mentioned techniques compare high-resolution products generated by processing images with SfM and MVS algorithms from acquired images in two different UAS photogrammetric survey epochs [22,23]. In the entire SfM-MVS algorithm, processing using the MVS algorithm is a significantly more time-consuming and demanding part of the processing algorithm [34,35,36].

In [37], we proposed a new data processing method for the calculation of the landslide displacements from UAS photogrammetric survey data based exclusively on the SfM algorithm steps and resulting sparse point clouds. Using the proposed method, landslide displacements can be determined without using the MVS algorithm and generating dense point clouds, digital elevation model, and digital orthomosaic images. Based on the aforementioned information and compared to previously used methods for detecting landslide displacement, the proposed data processing method is faster, computationally simpler, and does not compromise the quality of the obtained results.

The applicability of the proposed method is presented in [37], where simulated displacements are determined from the UAS survey performed on a test field at a flight altitude of 20 m. In this paper, the continuation of this research is presented using data from UAS surveys performed on the same established test field at greater flight altitudes (50 and 120 m) since they are more realistic altitudes for UAS survey of a landslide. Moreover, UAS surveys were performed by applying two different flight heights to analyze the impact of flight altitude on the accuracy of the determined displacement. On the established test field, displacements were determined using the proposed method and compared with the true value of displacements to show the accuracy of the proposed method. Furthermore, the determined displacements are compared with the displacements determined based on the comparison of orthomosaic images from the two epochs, which is the most commonly used method of determining displacements from the UAS photogrammetric survey.

Since the UAS surveys on an established test field were performed in ideal conditions on a relatively small and predominantly horizontal area with clearly defined details, the applicability of the proposed data processing method was also analyzed on the larger area of an actual landslide with complex topography. For this purpose, data from two UAS photogrammetric surveys of Kostanjek landslide were processed using the proposed data processing method. For the accuracy analysis of landslide displacement determination achieved using the proposed data processing method, in the case of the test field and an actual landslide, displacements were measured via classical geodetic surveying techniques using the total station and GNSS instruments of the Kostanjek landslide real-time monitoring system.

## 2. Materials and Methods

The workflow of the proposed data processing method with a novel approach for the calculation of landslide displacements from UAS photogrammetric survey data is described in Section 2.1. The test field established for the testing of the proposed method with mission planning parameters and data acquisition procedures necessary for the successful execution of the survey on the test field are described in Section 2.2. Section 2.3 describes landslide Kostanjek, as well as UAS photogrammetric surveys conducted to verify the performance of the proposed data processing method.

### 2.1. Workflow of the Proposed Data Processing Method

**Figure 1:**
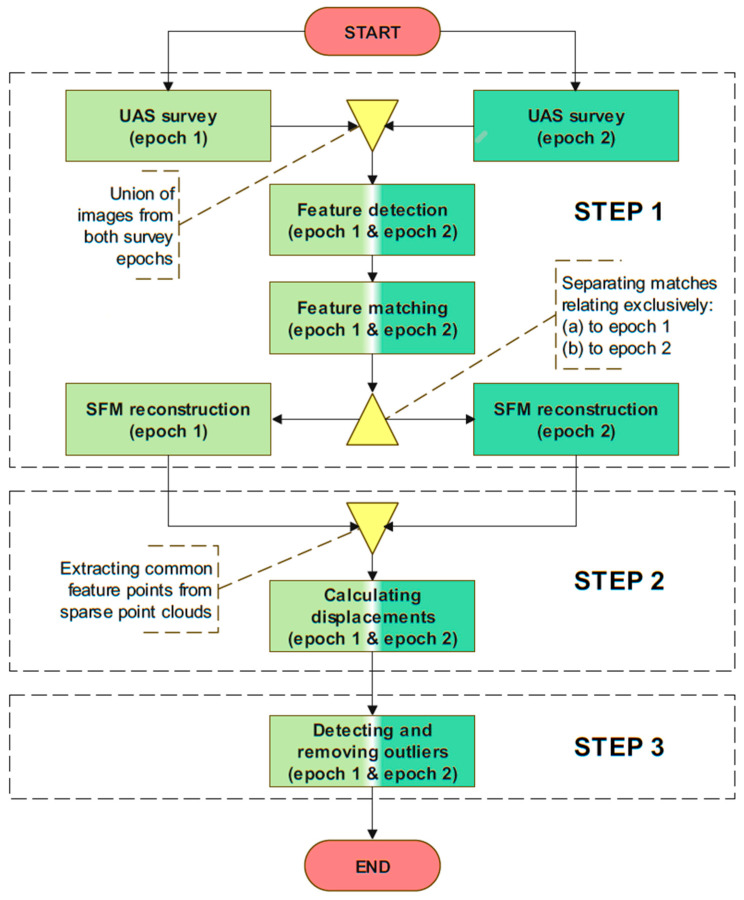
The flowchart of the proposed data processing method [37].

The theory behind the proposed data processing method is presented in detail in [37,38]. The workflow of the proposed method with three main steps is shown in Figure 1. In this section, those main steps of the proposed method will be briefly explained for the purpose of understanding and analyzing the results presented in this paper.

Within Step 1 of the proposed method, an algorithm for feature detection is applied to all images acquired during two distinct UAS survey epochs. After the features have been identified on all images from both UAS survey epochs, they are linked using feature matching algorithms. The Fast Library for Approximate Nearest Neighbors (FLANN) method of matching is used in this paper. Using the proposed method, it is necessary to perform a matching algorithm process between features on all images from both surveying epochs simultaneously. This will make it possible to connect the reconstructed points from two different surveying epochs that were processed separately and determine the displacements between them. After the features are matched, reconstruction of the scene using the SfM algorithm is performed separately for the first and second surveying epochs. This process led to the creation of two sparse point clouds where each point cloud consists of reconstructed point features that relate exclusively to the first and second epochs of the UAS survey, respectively. These sparse point clouds are rebuilt in local coordinate systems that must be connected with the same global coordinate system by means of an indirect or direct georeferencing technique. In this paper, we used an indirect georeferencing approach by using Ground Control Points (GCPs) established on the field. By obtaining two georeferenced sparse point clouds relating to an identical reference coordinate system, Step 1 of the workflow ends, and the calculation of displacement vectors follows.

Within Step 2, displacements are calculated from two sparse point clouds. The results of Step 1 are two georeferenced sparse point clouds that refer to two different epochs in an identical reference coordinate system. Numerous reconstructed feature points correspond to the same physical points in both epochs. It is straightforward to identify these points since features are matched simultaneously on images of both surveying epochs. Then, the displacement vectors can be calculated as the difference between coordinates in the two surveying epochs:

(1) $${\Delta E}_{i}=E_{i}(e_{2})-E_{i}(e_{1}),\;{\Delta N}_{i}=N_{i}(e_{2})-N_{i}(e_{1}),\;{\Delta H}_{i}=H_{i}(e_{2})-H_{i}(e_{1})$$

where:

${\Delta E}_{i},\;{\Delta N}_{i},\;{\Delta H}_{i}$—is the displacement in the east, north, and height directions,
i—is the identification name of the common feature point,
$e_{1},\;e_{2}$—denote the first and second epochs.

Within Step 3, filtering of the data and detecting and removing outliers from the data are performed. Filtering of the data was conducted based on the criteria of the number of images on each feature point from which the displacements were calculated. The reliability of the feature reconstruction increases with the number of images on which they have been detected. In this paper, only the displacement vectors calculated based on common feature points found on five and more images in both epochs were kept in the datasets. In the next step, outlier displacement vectors are detected and removed by performing the Leave One Out Cross Validation (LOOCV) process based on the kriging interpolation [39,40]. The detailed process of detecting and removing outlier displacements is described in [37].

As a result of this process, the number of displacement vectors in the dataset decreased by keeping the most reliable displacement vectors within the area of the landslide.

### 2.2. Test Field—Establishment and Simulation of the Landslide

**Figure 2:**
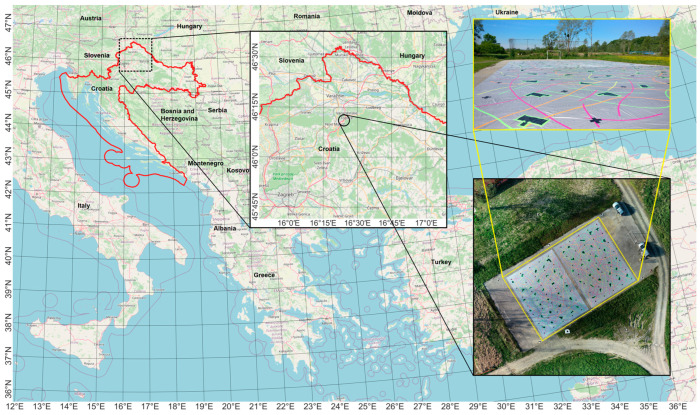
Location of the test field with a simulated landslide.

To examine the applicability and accuracy of a proposed data processing method in determining the displacements of a landslide, a test field on which the UAS photogrammetric surveys were carried out was established. A test field was established in the Republic of Croatia near village Ljubešćica (Figure 2).

A flat concrete surface of a football pitch measuring 40 m × 25 m was used as a surface for simulating landslide movements. The landslide was simulated by moving two large tarpaulins (dimensions 20 m × 15 m = 300 m^2^) placed on the football pitch. The tarpaulins were marked with random patterns and lines drawn on each of them (Figure 2).

**Figure 3:**
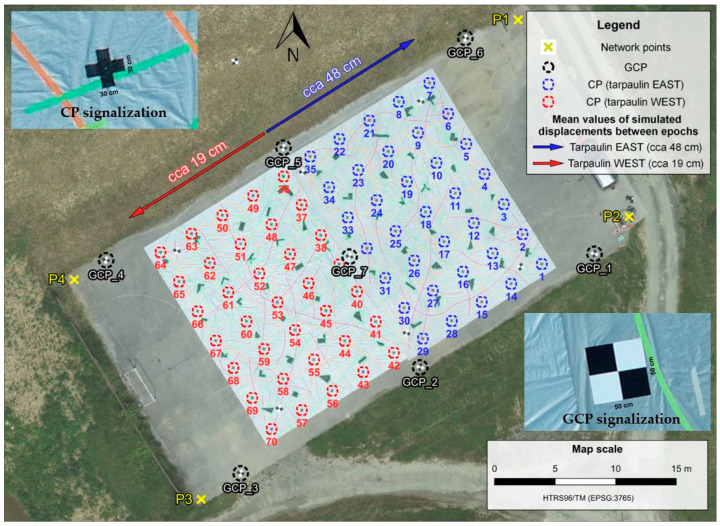
Presentation of the test field with mean tarpaulins displacements between epochs, and the distribution of network reference points, CPs, and GCPs.

The tarpaulins were moved in different directions and by different magnitudes relative to the initial positions. The first epoch (epoch 1) in this research represents the initial position of the tarpaulins, which means the state of the landslide before the simulated displacement, and the second epoch (epoch 2) denotes the state of tarpaulins after the simulated displacements. The average magnitude of the simulated displacements for the tarpaulin in the west was 19 cm, and for the tarpaulin in the east was 48 cm, as shown in Figure 3.

To validate the accuracy of the proposed data processing method, 70 control points (CPs) have been marked on tarpaulins (35 CPs on each tarpaulin) in a raster grid with a cell size of 3 m. The CPs were marked as a black cross 30 cm wide (Figure 3). Moreover, seven ground control points (GCPs) have been established on the test field for indirect georeferencing of UAS-derived products. The GCPs were marked as a flat square plate with 50 cm long sides on which the chessboard pattern was painted in black and white (Figure 3).

To determine the coordinates of CPs in each survey epoch as well as the coordinates of GCPs, the reference network was established. The reference network consisted of four points (points P1–P4 shown in Figure 3) stabilized in each corner of the football pitch. The measurements were performed at the Leica TPS1201 total station, with an angle measurement accuracy of 1″ and distance measurement accuracy of 2 mm + 2 ppm [41]. The final coordinates of reference network points were determined with sub-centimeter level accuracy.

After the test field was established, UAS missions containing all the flight parameters necessary for the successful execution of the survey on the test field were planned. Each planned mission was surveyed twice, first before simulating landslide displacements (epoch 1) and second after simulating displacements (epoch 2). The main difference between the two missions was the flight altitude of the UAS vehicle for the purpose of analyzing the impact of flight altitude on the accuracy of displacement determined using the proposed method. It is important to mention that the accuracy of the UAS survey mainly depends on the flight altitude [42].

Therefore, the first mission was prepared for a flight altitude of 50 m, and the second mission for a flight altitude of 120 m above ground level. The flight altitude of 120 m above ground level was chosen as the upper flight altitude limit because this altitude is the upper flying limit allowed in the open category prescribed by the official regulation on UAS in the European Union.

The missions were prepared for quadcopter vehicle DJI Phantom 4 Pro v2.0, with a built-in 1-inch, 20 Megapixels CMOS camera sensor. The detailed parameters of each UAS mission can be observed in Table 1.

Both missions, a total of four UAS photogrammetric surveys, were performed on 2 June 2021. The camera parameters (shutter speed and aperture) were manually adjusted before the flights, according to the light intensity, to obtain the best possible image quality of tarpaulins. The settings of the ISO parameter were set on automatic adjusting. The fly duration and camera parameters used during the performing mission can be seen in Table 2.

### 2.3. Case Study—Application of the Proposed Data Processing Method on the Kostanjek Landslide

**Figure 4:**
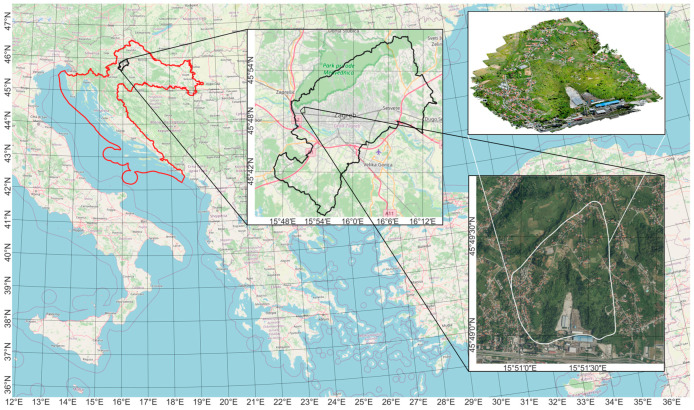
Location of the Kostanjek landslide.

The performance of the proposed data processing method was tested on the largest landslide in Croatia, the Kostanjek landslide, located in the western residential area of the City of Zagreb in the Republic of Croatia (Figure 4), at the base of the Medvednica Mountain [43]. The Kostanjek landslide is a deep-seated translational landslide that extends over an area of 1 km^2^ with an estimated volume of 32 million m^3^, with a sliding surface depth of up to 90 m, mainly composed of soft marls [8,44]. The location of the landslide is approximately between 45°48′57″ and 45°49′40″ latitudes and between 15°50′54″ and 15°51′40″ longitudes considering the WGS 84. According to the velocity landslide classes defined by [45], it is a slow-moving landslide with a maximum movement velocity of 44 cm/year, detected in the period from 1974 to 1976 [46]. From 1963 to 1994, the total displacements ranged from 3.4 to 6.5 m [44].

**Figure 5:**
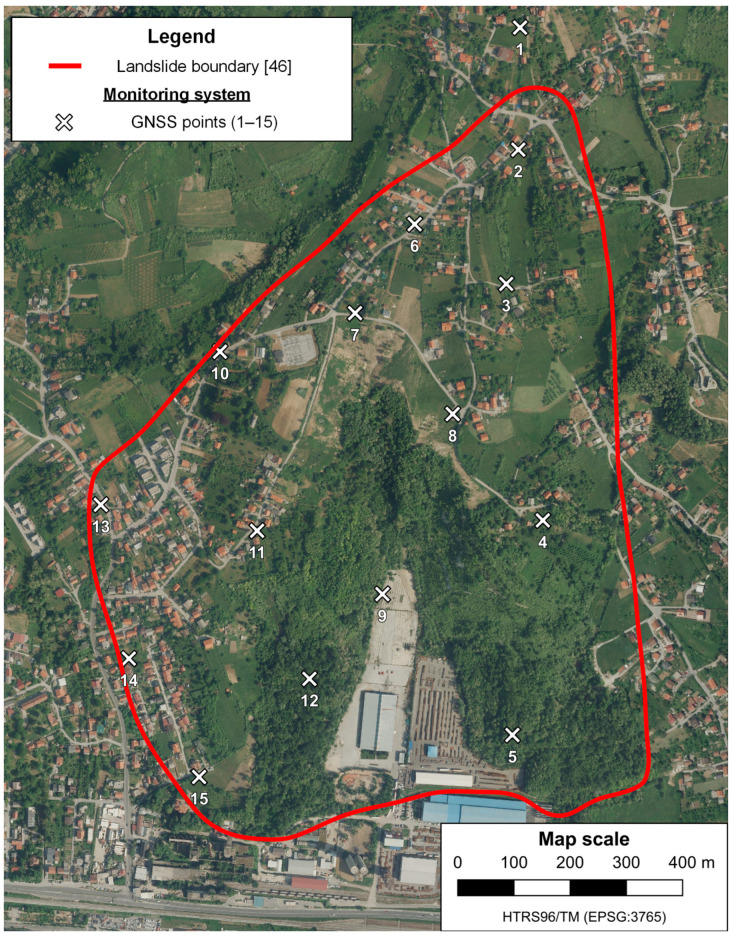
Locations of the permanently established GNSS sensors on the landslide for kinematics monitoring in real-time.

This landslide is an excellent choice for testing the applicability of the proposed data processing method in the determination of displacements on an actual landslide since it has an integrated automated real-time monitoring system (Observatory for the Kostanjek landslide monitoring). With the real-time monitoring system, it is possible to gain insight into the actual displacements of the Kostanjek landslide in a certain period that are used as reference displacement values during the process of validating the proposed data processing method. The actual landslide displacements are determined by using the monitoring system in real-time based on the 15 GNSS sensors permanently established on the landslide (Figure 5).

The reference values of displacement vectors at the position of the GNSS monitoring system sensors were determined by calculating the difference between their positions in two epochs. These GNSS sensors were used as CPs in the validation process. The position of the GNSS sensors in both epochs is determined based on the measurements of the real-time monitoring system “Observatory for the Kostanjek landslide monitoring.”

The images captured in UAS photogrammetric surveys on 24 April 2017, and 2 May 2019, were processed using the proposed data processing method. UAS missions were planned using the senseFly eMotion 3 software, senseFly Ltd, Cheseaux-sur-Lausanne, Switzerland. The parameters of each planned mission can be seen in Table 3.

UAS photogrammetric survey was conducted using a fixed wing vehicle senseFly eBee X. The difference between the two planned missions was in the used cameras and flight altitudes. In the first mission, the camera model was senseFly S.O.D.A, with a 1-inch 20-megapixel RGB sensor, and in the second mission, the camera model was senseFly Aeria X, with a 24-megapixel RGB APS-C sensor. Moreover, due to changes in legal regulations in the Republic of Croatia in the period between the two UAS surveys, UAS flight altitudes in the second mission was 40 m lower. All this led to smaller GSD values in the second mission (GSD in the first mission was 3.9 cm/px, and in the second mission, it was 2.3 cm/px).

A total of 562 images were obtained in the first mission, and a total of 1530 images were obtained in the second mission. The camera parameters used during the missions are shown in Table 4.

In both survey epochs on the Kostanjek landslide, 20 GCPs were established for indirect georeferencing, marked with a 20 cm diameter white circle and a survey bolt in the middle used for precise determination of GCP coordinates. The coordinates of GCPs were determined by means of the GNSS RTK method using the CROPOS differential correction services [47]. Measurements were conducted in two independent repetitions (each repetition consists of 3 consecutive measurements, each lasting 30 s) with a time interval between the repetitions of 2 h allowing for change in GNSS satellite geometry.

## 3. Results

In this section, the results of processing of the collected UAS photogrammetric survey data and accuracy analysis of displacements determination by the proposed data processing method are presented. Results of application of the proposed data processing method on the test field are presented in Section 3.1, with the accuracy analysis presented in Section 3.2. Results of the comparison of displacements determined by using the proposed data processing method and by the comparison of orthomosaic images from the two epochs are presented in Section 3.3. In Section 3.4, results of application of the proposed data processing method on an actual landslide are presented.

### 3.1. Test Field—Results of the Proposed Data Processing Method

Following the proposed data processing method workflow (Figure 1), the first step in image processing was to detect features on the images acquired in both survey epochs of the two UAS missions. The images were processed using the open-source OpenSfM v0.4.0 software [48]. The images of both epochs related to the same mission are processed together.

During the first mission, a total of about 47.89 million features were found across all 433 images, which resulted in a mean of 110.61 thousand features for each image. In addition, during the second mission, a total of about 34.04 million features were found across all 322 photos, which resulted in a mean of 105.72 thousand features for each image.

The next step of the proposed data processing method workflow is to match features between images acquired during UAS photogrammetric survey epochs. In this step, all possible matches between feature points on all images participating in the processing are found. This includes searching the matches between feature points on images from the first epoch, the matches between feature points on images from the second epoch, and most importantly for the proposed data processing method, searching the matches between feature points on the images of both epochs. In other words, the matches between features on two images are crucial, where one image is being from the first and the other image is being from the second epoch, since they will be used in the calculation of the displacements between two epochs using the proposed data processing method. Those matches are called common feature matches.

In the first mission, there were approximately 38.09 million matches detected between the features on images, out of which approximately 3.86 million (10.1%) are common. Furthermore, in the second mission, there were approximately 48.39 million matches detected between the features on images, out of which approximately 5.50 million (11.37%) are common matches.

After the matches between images had been found, the matching feature points were defined as a unique point that represents a set of feature points from different images that have been recognized to correspond to the same physical point. As a result, the total amount of all matching feature points in the first mission equals 2,151,475, while 60,935 relates to common feature points between epoch 1 and epoch 2. The ratio between common feature points to all matching feature points was 2.83%, meaning that it is possible to determine the displacements on approximately every 35th matching feature point. Furthermore, the total amount of all matching feature points in the second mission equals 2,038,643, whereas 72,531 relate to the common feature points between epochs. The ratio between common feature points to all matching feature points is 3.56%, meaning that it is possible to determine the displacements on approximately every 28th matching feature point.

The next step of the proposed data processing method workflow is SfM reconstructions, which were executed independently for each epoch in each mission, resulting in a total of four reconstruction processes. Therefore, for each mission, one reconstruction was based solely on the matches between the images from the first epoch (before simulating displacements), and the other was exclusively based on the matches between the images from the second epoch (after simulating displacements). The reconstructed sparse point clouds were georeferenced indirectly based on the 7 GCPs, as described in Section 2.1.

**Figure 6:**
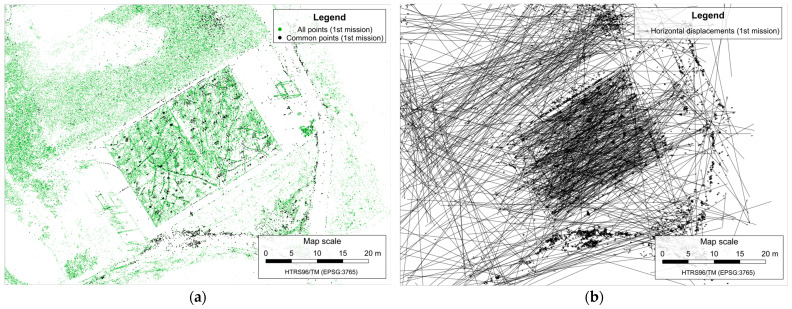
All reconstructed points with highlighted common feature points in the first mission (**a**), and determined horizontal displacements vectors (**b**).

**Figure 7:**
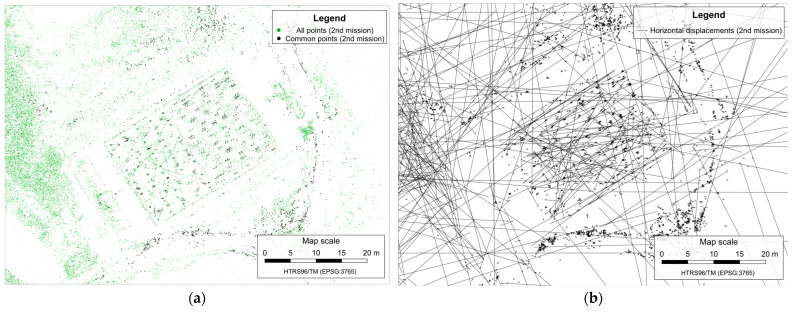
All reconstructed points with highlighted common feature points in the second mission (**a**), and determined horizontal displacements vectors (**b**).

After the SfM reconstructions, the number of common matching feature points in the first mission decreased from 60,935 to 16,339 (26.8%) (Figure 6a), and in the second mission decreased from 72,531 to 19,125 (26.4%) (Figure 7a).

The displacement determination is based on comparing the reconstructed sparse point clouds of the same mission from the two surveying epochs by calculating the differences between the coordinates of common matching feature points according to Equation (1). The horizontal displacement vectors determined between all common matching feature points in both missions are shown in Figure 6b and Figure 7b.

**Figure 8:**
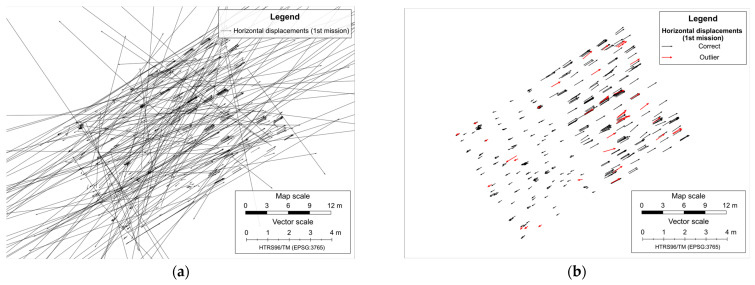
Remaining horizontal displacements vectors in the first mission after the filtering (**a**), correct and final displacement vectors with highlighted outliers in the first mission (**b**).

**Figure 9:**
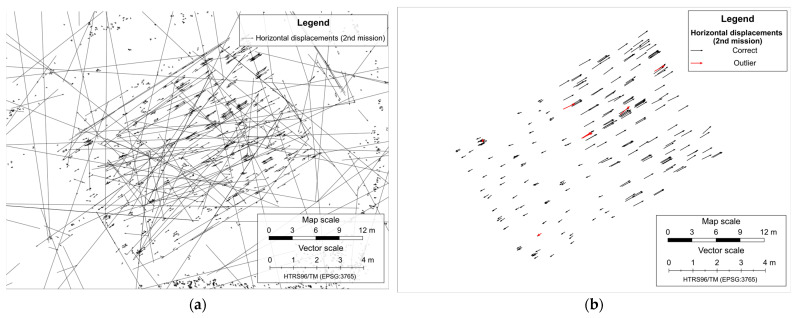
Remaining horizontal displacements vectors in the second mission after the filtering (**a**), correct and final displacement vectors with highlighted outliers in the second mission (**b**).

Before running the outlier removal process, the dataset is filtered keeping only the vectors between feature points found on at least five images in both epochs within each mission and located within the area of simulated landslide, resulting with reduction of the number of displacements vector from 16,339 to 722 (Figure 8a) in the first mission and from 19,125 to 304 (Figure 9a) in the second mission.

Before removing outlier displacement, additional filtering was performed to discard all displacement vectors whose 3D displacement magnitude is greater than a predefined threshold. In the case of the test field, this threshold was subjectively defined as 1 m, which was approximately twice the maximum magnitude of the simulated displacement between epochs. After performing this filtering, the total number of displacement vectors decreased from 722 to 526 in the first mission, and from 304 to 284 in the second mission.

When the dataset was filtered, removing the remaining outlier displacement vectors from datasets was conducted by performing the iterative LOOCV outlier-removing process based on the kriging interpolation method, as explained in Section 2.2.

After removing outliers, the total number of displacement vectors remaining was 482 in the first mission (Figure 8b) and 277 in the second mission (Figure 9b). The density of the remaining displacement vectors per square meter of simulated landslide area was 0.8 vector per m^2^ in the first mission and 0.46 vector per m^2^ in the second mission.

As it can be noticed from above-presented results, the number of displacement vectors in the dataset significantly decreased after performing the filtering of data, and detecting and removing outlier from data, but at the same time, the most reliable displacement vectors within the area of the simulated landslide were kept.

### 3.2. Test Field—The Accuracy of Displacement Determination Using the Proposed Data Processing Method

**Figure 10:**
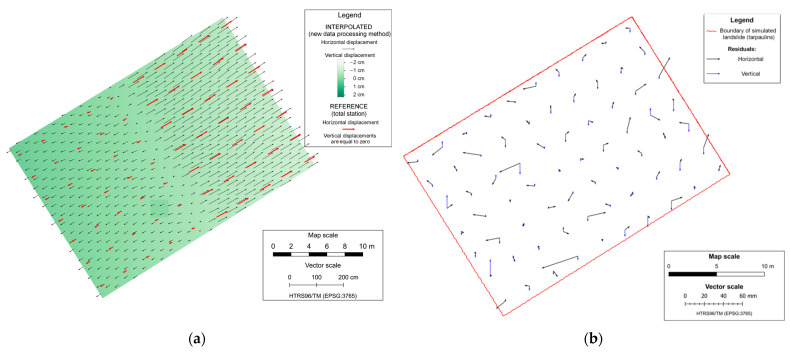
Displacement map of simulated landslide (**a**) and residuals in the first mission (**b**).

**Figure 11:**
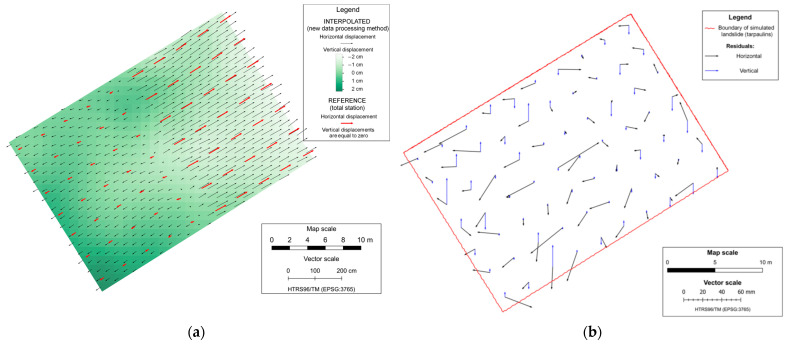
Displacement map of simulated landslide (**a**) and residuals in the second mission (**b**).

The remaining displacement vectors were used to generate a displacement map of simulated landslides for each mission, which was made by performing a kriging interpolation based on the datasets that consists of a displacement determined by the proposed data processing method. The interpolation was made on regular raster grid points with a cell size of 1 m, covering the area of the simulated landslide. The displacement maps of the simulated landslide are shown in Figure 10a and Figure 11a, where it can be seen that the displacements determined by using the proposed data processing method follow the reference displacements (obtained from the measurements with the total station) in magnitude and direction. Accuracy analysis of landslide displacement determination by using the proposed data processing method is performed by comparison of the displacement for all 70 CPs calculated from the total station measurements (which present referent displacement values) and determined displacement vectors of the proposed data processing method based on kriging interpolation with the origins in the same positions.

The range values of displacements in each mission in the east and north directions differ from zero (Table 5), indicating that the tarpaulins did not move uniformly along the entire surface. However, this is normal because it is impossible to move the entire tarpaulin manually on a flat surface and expect that the displacements over its entire surface at each point will be equal to each other. The reference displacement values in Table 5 indicate that the average horizontal displacement of the east tarpaulin was 48.2 cm in the north-east direction (59.0°). For the west tarpaulin, it was 18.9 cm in the south-west direction (242.4°). The average vertical displacement value for both tarpaulins indicates that they did not move in height (equal to zero). The determined displacement values are similar to the reference values in both missions. Thus, it can be seen that the mean determined vector values in both missions did not differ by more than 1.2 cm in magnitude and by more than 0.8° in the horizontal angle direction, and also did not differ by more than 0.2 cm in the vertical direction, compared to the reference values.

Displacement residuals are the difference between the reference value and the value of displacements determined using the proposed method (Table 6). The residuals range from −4.3 cm to 3.7 cm in the horizontal direction and from −2.8 cm to 1.8 cm in the vertical direction. Table 6 shows that the first mission, in which the UAS operated at a lower flight altitude and achieved more precise results, with mean values of residuals of 0.2 cm in the East direction, −0.1 cm in the North direction, and 0.2 cm in the vertical direction, compared to the results from the second mission, which had mean values of 0.6 cm in the East direction, 0.5 cm in the North direction, and 0.1 cm in the vertical direction. Figure 10b and Figure 11b provide the mission-specific graphical representations of the displacement residuals. The graphical representation of the displacement residuals for each mission can be seen in Figure 10b and Figure 11b, respectively.

The Root Mean Square Error (RMSE) is used as an indicator of the accuracy of displacement determination using the proposed data processing method. It represents the square root of the sum of the squares of the differences (residuals) between the determined (proposed data processing method) and reference (total station) coordinates of the identical CPs, and it is calculated for each coordinate direction by means of the following equations:

(2) $$RMSE\left(\Delta E\right)=\sqrt{\frac{\sum_{i=1}^{N}{\left[{\Delta E}_{i}-\hat{{\Delta E}_{i}}\right]}^{2}}{N}}\;RMSE(\Delta N)=\sqrt{\frac{\sum_{i=1}^{N}{\left[\Delta N_{i}-\hat{{\Delta N}_{i}}\right]}^{2}}{N}}\;RMSE(\Delta H)=\sqrt{\frac{\sum_{i=1}^{N}{\left[{\Delta H}_{i}-\hat{{\Delta H}_{i}}\right]}^{2}}{N}}$$

where:

${\Delta E}_{i},\;{\Delta N}_{i},\;{\Delta H}_{i}$—reference value,
$\hat{{\Delta E}_{i}},\;\hat{{\Delta N}_{i}},\;\hat{{\Delta H}_{i}}$—determined value,
*i*—index of the CP (i=1,…,70).

The summary of the achieved RMSE values of displacement determination by using the proposed data processing method for each coordinate direction, horizontal (2D) direction, and spatial (3D) direction for both UAS missions can be seen in Table 6.

The results in Table 6 indicate great accuracy of displacement determination with the proposed data processing method because in both missions, the RMSE value for all coordinate directions (1D) was maximally 1.4 cm. The accuracy of the determination of 2D displacements equals 1.0 cm in the first mission and 1.8 cm in the second mission. Furthermore, the accuracy of determination of 3D displacements equals 1.2 cm in the first mission, and 1.9 cm in the second mission, which indicates a good accuracy.

### 3.3. Test Field—Displacements Determined from Orthomosaics Images

After the displacements were determined using the proposed data processing method, the same displacements simulated on the test field were determined by using a commonly used method based on comparing manually tracked features of SfM-MVS derived orthomosaics from different epochs. This was performed to analyze the possibility to determine displacement vectors with the same accuracy using the proposed data processing method.

**Figure 12:**
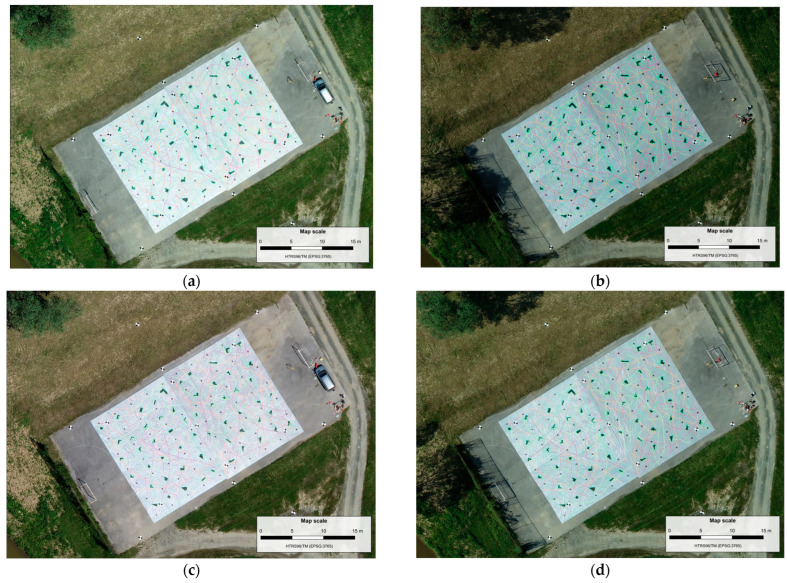
Orthomosaics of the test field in the first (**a**) and second (**b**) epoch of the first mission, and in the first (**c**) and second (**d**) epoch of the second mission.

Orthomosaic images are produced using OpenDronMap (ODM) v2.9.0 software [49] based on the sparse point clouds that are generated during the determination of the displacements using the proposed method. Since the surveys in two UAS missions were made twice in different time epochs (before and after simulating displacements), it resulted in the production of four orthomosaics (Figure 12).

The achieved GSD values of generated orthomosaics was 1.4 cm/px in the first mission and 3.3 cm/px in the second mission. The determination of simulated landslide displacements was performed based on comparing the manually tracked features on SfM-MVS-derived georeferenced orthomosaics resulting from different UAS survey epochs. When tracking the features manually, it is necessary that the chosen features on orthophotos be distinctly recognizable between the epochs. For this purpose, we used clearly recognizable cross marks on the tarpaulins where the center of the cross also marks the position of the CP (Figure 3). Each tracked feature on the orthomosaics denotes the center of the cross, and horizontal displacement vectors were calculated based on determining the coordinates and their differences between the epochs.

The accuracy of the method based on the comparison of orthomosaic images was performed by calculating displacement residuals as a difference between referent and displacement values determined from orthomosaic images (Table 7). Since both values denote the displacements between the center of the same cross marks (CPs) on the tarpaulins from different epochs, a comparison was conducted directly i.e., it was not necessary to perform kriging interpolation, as it was performed for calculating displacement residuals for displacements determined by using the proposed data processing method.

The residuals in the horizontal direction range between −5.4 cm to 7.5 cm, and from the results shown in Table 7, it can be seen that more precise results were achieved in the first mission with lower (50 m) flight altitude.

The results of the accuracy in determining the displacements from orthomosaic images indicate good accuracy, because in both missions, the RMSE value for each coordinate direction was lower than 2.3 cm. The RMSE of determining the horizontal displacements equals 2.2 cm in the first mission and 2.9 cm in the second mission. When compared to the achieved accuracy of the proposed data processing method (Table 6), it can be noticed that the simulated landslide displacements were determined more accurately by using the proposed data processing method. Moreover, it should be noted that the vertical displacement vectors can be determined using the proposed data processing method, which is not possible from orthomosaic images.

### 3.4. Case Study—The Accuracy of Displacement Determination Using the Proposed Data Processing Method on the Kostanjek Landslide

Processing of the captured images during two UAS photogrammetric surveys of the Kostanjek landslide using the proposed data processing method was carried out according to the steps described in Section 2.1. The first step in data processing was to detect features on images collected in both UAS survey epochs. The total number of 196,428,504 features were found on all 2092 images from both surveys, which gives an average of 93.85 thousand features detected per image. From the total number of found features, 23.81% of found features are related to the first epoch, and the other 76.19% are related to the second epoch. The ratio is 1 to 3.2, which is expected because almost three times more images were captured in the second epoch.

The next step of the proposed data processing method is feature matching. A total of 29.02 million matches are found among all images where over 2.19 million are common matches between epochs. The next step was the matching of the feature points. The total number of matching feature points equals to 7,529,936, while 675,485 of them are detected on images from both epochs.

**Figure 13:**
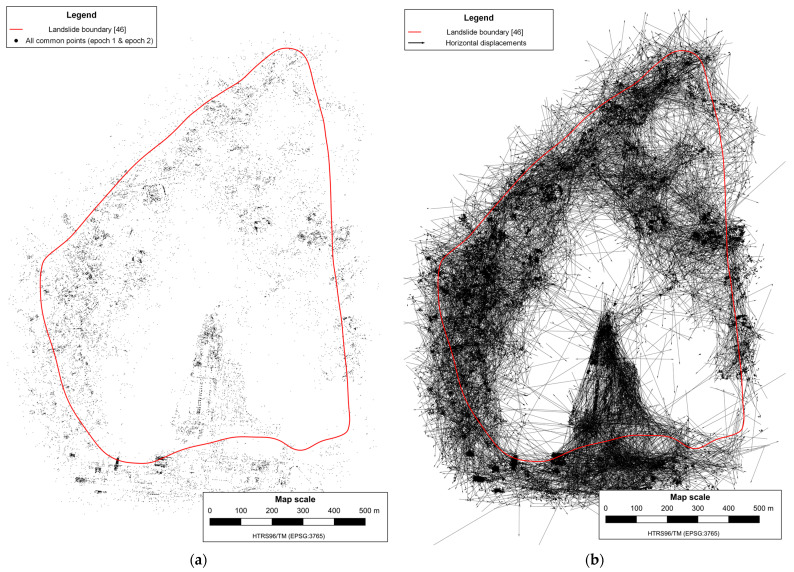
All reconstructed common feature points in both epochs (**a**), and determined horizontal displacements vectors (**b**).

After the feature matching process, SFM reconstructions were executed independently for each epoch. One reconstruction was based solely on the features and matches between images from the first epoch, and the other was exclusively based on features and matches between images from the second epoch. After the SfM reconstructions, the number of common matching feature points decreased from 675,485 to 33,545 (5.0%) (Figure 13a). The next step was calculating the displacements based on the comparison of reconstructed sparse point clouds from two survey epochs by calculating the differences between the coordinates of common matching feature points (Figure 13b) using Equation (1).

**Figure 14:**
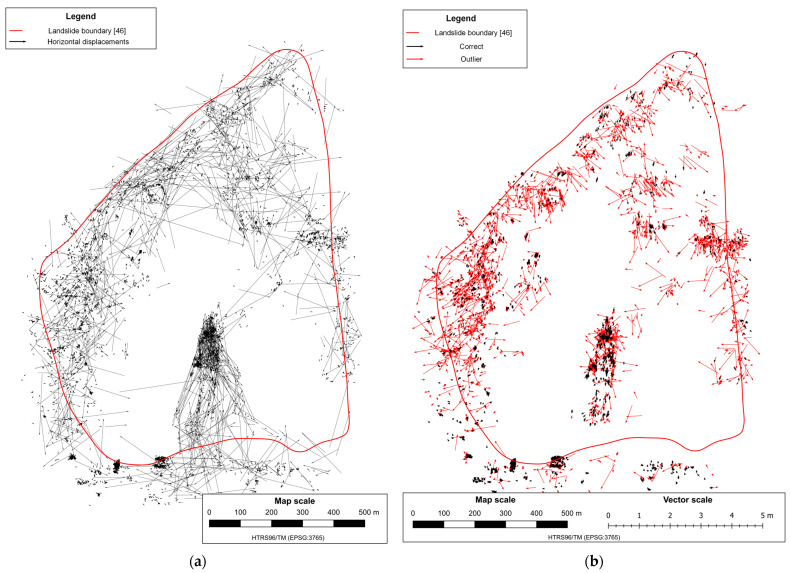
Remaining horizontal displacement vectors after the filtering (**a**), and final displacements vectors with highlighted outliers (**b**).

As explained in Section 2.1, the dataset is filtered before running the outlier removal process, keeping only the vectors between feature points found on at least five images in both epochs, resulting in the reduction of the number of displacements vector from 33,545 to 5657 (Figure 14a). After removing outlier displacement vectors (Figure 14b) from datasets by performing LOOCV processes based on the kriging interpolation method, the total number of displacement vectors that remained was 1822.

**Figure 15:**
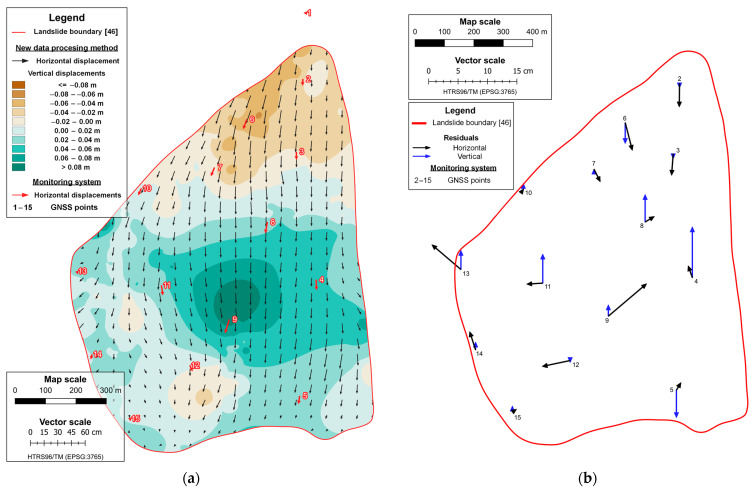
Displacement map of the Kostanjek landslide (24 April 2017–2 May 2019) (**a**) and residuals in 14 GNSS monitoring system points (**b**).

The displacement map of the Kostanjek landslide was prepared by performing kriging interpolation, where the interpolation was based on the displacement dataset that consists of a displacement determined by using the proposed data processing method. The points on which the displacement vectors were determined are the points of a regular raster grid covering the landslide area. The landslide map can be seen in Figure 15a, indicating that the determined displacements successfully follow the actual displacements in magnitude and direction (red vectors show actual displacements determined with the measurements of 15 GNSS monitoring system sensors).

The proposed data processing method is validated by comparing its determined displacement vectors with the displacement vectors determined in the measurements with the GNSS monitoring system sensors and their values are considered as reference values. In order to consider the comparison as valid, the vectors being compared must have their origin at an identical position. Since the origins of the reference displacement vectors refer to the positions of the GNSS sensor in the first epoch, it is necessary to determine the displacement vectors of the proposed data processing method based on kriging interpolation in the same positions. The kriging interpolation was used to predict displacement vectors at the position of the GNSS sensors based on the displacement dataset determined by means of the proposed data processing method. Thus, out of 15 GNSS sensors of the monitoring system of the Kostanjek landslide, 14 were used as CPs in the validation process. One GNSS point (GNSS 01) was placed outside the landslide area, and therefore, it was not used in the validation process. Displacement residuals are calculated as a difference between referent and displacement values determined by using the proposed method (Table 8).

The statistics results in Table 8 show that the residuals in the east direction ranged between −5.0 cm and 6.5 cm, with a mean value of −0.2 cm; and in the north direction, they ranged between −4.7 cm and 5.5 cm, with a mean value of 0.1 cm; and in height, they ranged between −4.7 cm and 8.7 cm, with a mean value of 1.2 cm. The graphical presentation of the displacement residuals is shown in Figure 15b.

According to the results shown in Table 8, it can be concluded that the displacement vectors are determined with reasonable accuracy since the RMSE values of the determined horizontal (2D) displacements equal 4.0 cm, and the RMSE value of the determined spatial (3D) displacements equals 5.4 cm. These accuracies represent an acceptable result since the flight altitude was higher by 40 m in the first epoch than in the second epoch, and the camera used for the acquisition of images in the first epoch had a shorter focal length than the camera in the second epoch, leading to a significant difference in image GSD values between the epochs (GSD(epoch 1) = 4.1 cm/px, and GSD(epoch 2) = 2.6 cm/px).

## 4. Discussion

The presented research deals with the applicability and accuracy of the method with a novel approach for calculating landslide displacements from UAS photogrammetric survey data, based exclusively on SfM algorithm steps without using the MVS algorithm. The workflow of the proposed method with its three main steps is elaborated, and the results of processing UAS photogrammetric survey images from an established simulated landslide and from the Kostanjek landslide are described by using a proposed method.

To assess the applicability of the proposed data processing method, two UAS missions at different flight altitudes (50 m and 120 m) were performed on the test field with simulated displacements. The displacements determined using the proposed data processing method were compared to the referent values determined by using total station measurements in 70 CPs on an established test field. The simulated displacements were within the range of 19 to 48 cm and were very precisely determined for accuracy analysis. The result presented in Section 3.2. proved that the proposed data processing method can successfully determine the spatial (3D) displacements of landslides with centimeter level accuracy even at a flight height of 120 m, although it is important to point out that the test field was established in a relatively small area, and therefore, better results were obtained than could be expected from a real landslide that extends over a larger area.

The proposed data processing method was also validated by comparing its results with the results obtained by a commonly used method based on comparing manually tracked features on orthomosaics from different epochs. The result presented in Section 3.3. showed that the simulated landslide displacements were determined more accurately by using the proposed data processing method than by using the method based on the comparison of orthomosaic images, by which it is additionally confirmed the applicability of the proposed data processing method and no need for additional processing of the images by using the MVS algorithm. Moreover, it should be noted that the vertical displacement vectors cannot be determined based on the comparison of orthomosaic images.

Since these results were obtained in ideal conditions on a relatively small test field established on a predominantly horizontal surface, the applicability of the proposed data processing method was further analyzed on the larger area of an actual landslide with significant height differences and complex topography. The determined displacements were compared with the reference values determined from the measurements of the GNSS real-time monitoring system.

On the Kostanjek landslide that extends over an area of 1 km^2^, the accuracy of determining the horizontal (2D) displacements was 4.0 cm, and that for the spatial (3D) displacements was 5.4 cm. The inferior results in accuracy were achieved compared to measurements on the test field. This is partly the result of different flight altitudes between the two UAS surveys (due to changes in legal regulations in the Republic of Croatia in the period between two UAS surveys) and the use of different cameras within survey epochs. Furthermore, the accuracy of determining the displacement is also affected by the distribution and positioning accuracy of established GCPs in the field, which was better in the case of the test field where the coordinates of the GCPs were determined more precisely by the total station measurements compared to the GNSS RTK method used on the Kostanjek landslide. Moreover, in the case of the test field, GCPs were better signalized i.e., they were better visible and recognizable in the acquired images, which resulted with more accurate georeferenced and refined sparse point clouds. Furthermore, in the case of the Kostanjek landslide, the terrain is covered with high vegetation, which resulted with areas without any matching feature points.

At the end, from elaborated accuracy analysis of determining displacements at the test field and the Kostanjek landslide, we can conclude that by using the proposed method, it is possible to detect displacements with a sub-decimeter level of magnitude. An advantage of the proposed method is the fact that it is not necessary to generate products such as dense point clouds, digital elevation models, and orthomosaics with the MVS algorithm that is more time-consuming than the SfM algorithm to detect displacements. This was confirmed by the achieved accuracy of determining the displacements from created sparse point clouds using only the SfM algorithm. Furthermore, since the SfM-MVS-derived products use a sparse point cloud as the basis for their creation, the errors in the sparse point cloud will propagate to all derived products. Then, all derived products (e.g., orthomosaics) will be influenced by those errors as well as errors introduced during the process of meshing or gridding. As a main disadvantage of the proposed method compared to the existing one, it can be pointed out that, in areas where there are no clearly defined feature points (e.g., forest, meadow, etc.), it would not be possible to determine displacements using our method, since it would not be possible to perform quality feature matching.

The most important steps of the proposed method are feature detecting and feature matching, both of which directly affect the accuracy, reliability, and number of determined displacements. Therefore, in our future work, improvements in these steps will be analyzed by using different types and methods or combining multiple methods of feature detection and feature matching in processing images to obtain better results.

## 5. Conclusions

This research presents the examination of a new approach for processing UAS photogrammetric survey data for the task of landslide monitoring. The proposed method is based on matching features between images from the UAS photogrammetric survey that were taken at two different times. This allows the calculation of displacements based only on the comparison of two reconstructed sparse point clouds, thus avoiding the need to produce dense point clouds, digital terrain models, or orthomosaic maps, all of which are produced via image processing with the MVS algorithm.

The results from the test field presented in this research proved that the proposed data processing method can successfully determine the spatial (3D) displacements of landslides with centimeter accuracy, which makes it suitable for the monitoring of landslide displacement. It can be concluded that the proposed data processing method offers a faster and computationally simpler way of calculating landslide displacement compared to previous methods, since it uses only the SfM algorithm to create sparse point clouds in different epochs. Thus, it can be avoided to generate products such as dense point clouds, digital elevation models, and orthomosaics with the MVS algorithm that is more time-consuming than the SfM algorithm.

Based on the results obtained on the test field and the Kostanjek landslide, future work on testing and improving the proposed data processing method is defined in the Discussion, aiming to improve the accuracy and reliability of determining displacements by using the proposed method.

## Figures and Tables

**Table 1:** The parameters of planned UAS missions.

Mission Parameters	First Mission	Second Mission
Number of waypoints	38	50
Approximate flight area [m^2^]	11,000	44,000
Distance between consecutive images [m]	10.94	25.97
Tilt of camera	70°	
Forward overlap	80%	
Side overlap	85%	
Flying altitude (above ground level) [m]	50	120
Flying speed [m/s]	2.5	5
Ground sample distance (GSD) [cm/px]	1.50	3.56

**Table 2:** Summary of UAS missions and camera parameters.

	First Mission		Second Mission	
	Epoch 1	Epoch 2	Epoch 1	Epoch 2
Start time [hh:mm]	11:46	16:24	10:42	15:37
End time [hh:mm]	12:02	16:41	10:58	15:52
Flight duration [hh:mm]	0:16	0:17	0:16	0:15
Number of acquired images	217	216	161	161
Shutter speed	1/400			
Aperture	8			
ISO (auto)	100			

**Table 3:** The parameters of planned UAS missions in senseFly eMotion 3 software.

Mission Parameters	First Mission	Second Mission
Number of waypoints	56	82
Approximate surveyed area	1.619 km^2^	1.624 km^2^
Distance between consecutive images	43 m	22 m
Tilt of camera	90°	90°
Forward overlap	70%	80%
Side overlap	75%	70%
Flying altitude (above ground level)	160 m	120 m
Flying speed	10 m/s	11 m/s
Ground sample distance	3.9 cm/px	2.3 cm/px

**Table 4:** Summary of accomplished missions with camera parameters.

	UAS Missions	
	First Mission (24 April 2017)	Second Mission (2 May 2019)
Start time [hh:mm]	9:51	9:20
End time [hh:mm]	10:36	10:25
Flight duration [hh:mm]	0:45	1:05
Number of acquired images	562	1530
Shutter speed (auto)	1/1000	1/2000
Aperture (auto)	F/2.8–F/7.1	F/3.6–F/5.6
ISO (auto)	125–320	160–640

**Table 5:** The statistics of the determined (proposed data processing method) and the referent (total station) displacements vector values for each tarpaulin in both missions.

Statistics Values	Tarpaulin in the East (n = 35)			Tarpaulin in the West (n = 35)		
	∆E [cm]	∆N [cm]	∆H [cm]	∆E [cm]	∆N [cm]	∆H [cm]
	Referent (total station) displacements					
Min	39.6	21.7	−0.4	−19.0	−11.6	−0.2
Max	43.7	28.0	0.3	−13.3	−4.9	0.4
Range	4.1	6.3	0.7	5.7	6.7	0.6
Mean	41.3	24.9	0.0	−16.8	−8.8	0.1
Mean 2D	48.2 cm, 59.0° NE direction			18.9 cm, 242.4° SW direction		
	Determined displacements—First mission					
Min	38.6	21.9	−1.1	−20.6	−11.8	−1.0
Max	44.8	29.0	0.8	−14.0	−4.9	2.0
Range	6.3	7.1	1.9	6.6	6.8	3.1
Mean	41.4	25.2	−0.4	−17.3	−8.9	0.1
Mean 2D	48.4 cm, 58.7° NE direction			19.7 cm, 242.8° SW direction		
	Determined displacements—Second mission					
Min	37.9	21.0	−1.2	−21.2	−12.5	−1.7
Max	44.1	28.0	0.8	−11.8	−4.6	2.8
Range	6.1	7.0	2.0	9.4	7.9	4.5
Mean	41.1	24.6	−0.5	−17.7	−9.5	0.3
Mean 2D	47.9 cm, 59.1° NE direction			20.1 cm, 241.6° SW direction		

**Table 6:** Statistics of residuals between determined and referent displacement vectors values and RMSE of determining displacements with the proposed data processing method.

	Residuals in the First Mission [cm]			Residuals in the Second Mission [cm]		
	∆E	∆N	∆H	∆E	∆N	∆H
Min	−1.9	−2.0	−1.8	−4.3	−2.7	−2.8
Max	3.7	1.2	1.2	3.4	3.0	1.8
Range	5.6	3.2	3.0	7.7	5.7	4.6
Mean	0.2	−0.1	0.2	0.6	0.5	0.1
RMSE 1D	0.9	0.5	0.6	1.4	1.1	0.8
RMSE 2D	1.0		-	1.8		-
RMSE 3D	1.2			1.9		

**Table 7:** Statistics of residuals between the referent displacement vectors and vectors determined from orthomosaic images and RMSE of determining displacements from orthomosaic images.

	Residuals in the First Mission [cm]		Residuals in the Second Mission [cm]	
	∆E	∆N	∆E	∆N
Min	−4.4	−4.0	−5.4	−5.4
Max	3.2	2.8	7.5	3.9
Range	7.6	6.8	12.8	9.3
Mean	−0.3	−0.4	0.0	−0.4
RMSE 1D	1.7	1.4	2.3	1.7
RMSE 2D	2.2		2.9	

**Table 8:** Statistics of residuals between the determined and referent displacement vectors values and RMSE of determining displacements with the proposed data processing method.

Statistics Values	Residuals (n = 14)		
	∆E [cm]	∆N [cm]	∆H [cm]
Min	−5.0	−4.7	−4.7
Max	6.5	5.5	8.7
Range	11.5	10.3	13.4
Mean	−0.2	0.1	1.2
RMSE 1D	2.7	3.0	3.5
RMSE 2D	4.0		
RMSE 3D	5.4		

## Data Availability

The data presented in this study are available on request from the corresponding author.
